# High exposure to hepatitis C virus in Saravan, southern Laos; identification of several risk practices

**DOI:** 10.1016/j.lanwpc.2022.100632

**Published:** 2022-11-07

**Authors:** Antony P. Black, Siriphone Virachith, Vilaysone Khounvisith, Lisa Hefele, Phimpha Paboriboune, Judith M. Hübschen

**Affiliations:** a Lao Lux Laboratory, Institut Pasteur du Laos, Vientiane Capital, Laos; b Department of Infection and Immunity, Luxembourg Institute of Health, Esch-sur-Alzette L-4354, Luxembourg; c Centre d'Infectiologie Lao-Christophe Mérieux, Vientiane Capital, Laos

Our previous hospital-based study in Saravan province, southern Laos, identified a large proportion of individuals who were seropositive for antibodies against hepatitis C virus (HCV). The majority were from Samuoi district where more than 40% of participants aged over 30 years had anti-HCV antibodies.^1^ These data contrasted to the low seroprevalence found in Lao blood donors, female garment factory workers and healthcare workers from other provinces.^2^, ^3^, ^4^

With the aim of assessing the risk practices within the community, we returned to Samuoi district from January until March 2022. In order to maximise the number of exposed participants recruited into the study, we selected 24 villages with previously identified seropositive individuals aged 18 or over. When possible, these individuals were recruited into the study and age-matched participants (±5 years) were randomly selected from the village name list. All selected participants completed a structured face-to-face questionnaire to identify risk practices and were then tested for anti-HCV antibodies by a World Health Organization pre-qualified rapid test (SD Bioline; 99.3% sensitive and 98.1% specific). Participants were classed as cases if seropositive and controls were seronegative. In the event of newly identified cases, more age-matched individuals were enrolled.

Participants (n = 402) were aged 20–90 years and 60.2% were male. There were 160 (39.8%) cases including 18 from the previous study and 242 controls. 94/160 (58.8%) of the cases had detectable viral load (Abbott Real-time HCV assay) and were referred to the district hospital for clinical assessment and treatment (all testing and treatment protocols were according to WHO and national guidelines and were provided free of charge). The average age was similar for RNA positive (50 years) and negative (49.4 years) cases.

Overall, there was a high number of reports of having received parenteral medication (58.0%) from non-licenced practitioners and healthcare workers in public health facilities. Use of re-used syringes was high (72.5% of those that reported receipt of parenteral medication), both by non-licenced practitioners and from the local health care facilities (until 20–25 years ago; personal communication, Dr. Bounluay Suaymanivong, director of the District Health Office; 8 February 2022).

Tattooing was frequent (33.1%) and reportedly non-sterile, including sharing of equipment with friends. Piercing was also common (95.0% of women) and non-sterile. Accidental blood exposure was reported by 37.3% of all participants e.g. when helping others who were involved in accidents.

Having tattoos (adjusted odds ratio (aOR), 1.81, 95% confidence intervals (1.2, 2.8), p = 0.01) and accidental blood exposure (aOR 1.9 (1.2, 2.9), p < 0.01) were significantly associated with anti-HCV seropositivity (Table 1). Viral sequencing is ongoing to determine the epidemiology of the infection in more detail.

**Table 1:** Characteristics and risk factors of participants.

Participant characteristics		Overall; N = 402; n (%)	Controls; N = 242; n (%)	Cases; N = 160; n (%)	aOR^a^ [95%CI]	p value
Sex	Male	242 (60.2)	128 (52.9)	114 (71.3)	ref	
	Female	160 (39.8)	114 (47.1)	46 (28.8)	0.51 [0.3, 0.8]	**<0.01**
Marital status	Married	370 (92.0)	219 (90.5)	151 (94.4)	ref	
	Single/divorced/widowed	32 (8.0)	23 (9.5)	9 (5.6)	0.6 [0.3, 1.4]	0.23
Ethnicity	Pako	378 (94.0)	221 (91.3)	157 (98.1)	ref	
	Other	24 (6.0)	21 (8.7)	3 (1.9)	0.2 [0.1, 0.70]	**0.01**
Has children	Yes	380 (94.5)	225 (93.0)	155 (96.9)	ref	
	No	22 (5.5)	17 (7.0)	5 (3.1)	0.66 [0.2, 1.9]	0.45
Education	No schooling	168 (41.8)	99 (40.9)	69 (43.1)	ref	
	Elementary school	141 (35.1)	78 (32.2)	63 (39.4)	0.76 [0.5, 1.3]	0.28
	Secondary or higher	93 (23.1)	65 (26.9)	28 (17.5)	0.48 [0.3, 0.9]	**0.02**
Profession	Unemployed	131 (32.6)	69 (28.5)	62 (38.8)	ref	
	Housewife/housekeeper	102 (25.4)	70 (28.9)	32 (20.0)	1.2 [0.6, 2.6]	0.65
	Farm worker	126 (31.3)	73 (30.2)	53 (33.1)	0.81 [0.5, 1.4]	0.43
	Other	43 (10.7)	30 (12.4)	13 (8.1)	0.52 [0.2, 1.1]	0.10
Income	<100 USD	354 (88.1)	207 (85.5)	147 (91.9)	ref	
	100-300 USD	48 (11.9)	35 (14.5)	13 (8.1)	0.6 [0.3, 1.2]	0.15
Had tooth filing^b^	No	364 (90.6)	219 (90.5)	145 (90.6)	ref	
	Yes	38 (9.5)	23 (9.5)	15 (9.4)	0.7 [0.3, 1.5]	0.37
Had surgery	No	367 (91.3)	219 (90.5)	148 (92.5)	ref	
	Yes	35 (8.7)	23 (9.5)	12 (7.5)	0.82 [0.4, 1.8]	0.62
Used traditional medicine	No	242 (60.2)	156 (64.5)	86 (53.8)	ref	
	Yes	160 (39.8)	86 (35.5)	74 (46.3)	1.36 [0.9, 2.1]	0.15
Received parenteral medicine	No	169 (42.0)	111 (45.9)	58 (36.3)	ref	
	Yes	233 (58.0)	131 (54.1)	102 (63.8)	1.2 [0.8, 1.9]	0.43
Received dental care	No	346 (86.1)	207 (85.5)	139 (86.9)	ref	
	Yes	56 (13.9)	35 (14.5)	21 (13.1)	0.79 [0.4, 1.4]	0.45
Received blood transfusion	No	396 (98.5)	239 (98.8)	157 (98.1)	ref	
	Yes	6 (1.5)	3 (1.2)	3 (1.9)	1.19 [0.2, 6.5]	0.84
Has tattoo	No	269 (66.9)	176 (72.7)	93 (58.1)	ref	
	Yes	133 (33.1)	66 (27.3)	67 (41.9)	1.81 [1.2, 2.8]	**0.01**
Has piercings	No	230 (57.2)	123 (50.8)	107 (66.9)	ref	
	Yes	172 (42.8)	119 (49.2)	53 (33.1)	1.21 [0.5, 2.8]	0.65
Has a war wound	No	362 (90.1)	221 (91.3)	141 (88.1)	ref	
	Yes	40 (10.0)	21 (8.7)	19 (11.9)	0.58 [0.3, 1.3]	0.17
Has chronic disease	No	171 (42.5)	107 (44.2)	64 (40.0)	ref	
	Yes	84 (20.9)	50 (20.7)	34 (21.3)	1.2 [0.7, 2.1]	0.52
	Don't know	147 (36.6)	85 (35.1)	62 (38.8)	1.4 [0.9, 2.3]	0.15
Accidentally exposed to blood	No	252 (62.7)	170 (70.3)	82 (51.3)	ref	
	Yes	150 (37.3)	72 (29.8)	78 (48.8)	1.9 [1.2, 2.9]	**<0.01**
Someone in family infected with hepatitis or HIV	No	373 (92.8)	225 (93.0)	148 (92.5)	ref	
	Yes	29 (7.2)	17 (7.0)	12 (7.5)	1.07 [0.5, 2.4]	0.86

Subset: Participants who received parenteral medication						
		Overall; N = 233; n (%)	Controls; N = 131; n (%)	Cases; N = 102; n (%)	aOR^a^ [95%CI]	p value
Needles were reused	No	64 (27.5)	46 (35.1)	18 (17.6)	ref	
	Yes	169 (72.5)	85 (64.9)	84 (82.4)	2.7 [1.4, 5.1]	**<0.01**
Who administered parenteral medication	Health care workers	16 (6.9)	10 (7.6)	6 (5.9)	ref	
	Other	114 (48.9)	72 (55.0)	42 (41.2)	1.88 [1.1, 3.3]	**0.03**
	Don't know	103 (44.2)	49 (37.4)	54 (52.9)	0.81 [0.3, 2.5]	0.7

A pre-tested questionnaire was administered by the research team, with translation into the local dialect by a trained healthcare worker when necessary. All data were analysed in R. The adjusted odds ratio for being anti-HCV antibody positive was the main outcome measure. There were no reports of intravenous drug use, dialysis or men who have sex with men. All participants had to be aged 18 or over to be included in the study. Significant p values (<0.05) are indicated in bold.

95% CI = 95% confidence interval; aOR = adjusted odds ratio; N = total number; n = number; USD = United States Dollars.

a Adjusted for sex and age. No adjustment for multiplicity has been performed.

b This traditional practice has largely ceased but, according to the participants, was common among adolescents up until approximately the 1970s.

This study highlights the urgent need to carry out extensive community-based testing for exposure and ongoing infection and treatment for HCV and possibly also other blood-borne infections in Saravan and also neighbouring provinces in Laos and Vietnam, which might have a similar burden of HCV infection. Community and healthcare worker awareness campaigns are needed, focusing on identified transmission risk practices, how to mitigate them, disease consequences and importance of treatment.

## Contributors

Antony P. Black: conceptualisation, funding acquisition, investigation, methodology, project administration, supervision, writing – original draft, and writing– review & editing. Siriphone Virachith: conceptualisation, data curation, formal analysis, investigation, methodology, project administration, writing– review & editing. Vilaysone Khounvisith: conceptualisation, methodology. Lisa Hefele: formal analysis, writing– review & editing. Phimpha Paboriboune: conceptualisation, funding acquisition, investigation, methodology, supervision, project administration, writing – review & editing. Judith M. Hübschen: conceptualisation, funding acquisition, methodology, resources, supervision, writing – original draft, and writing– review & editing.

## Declaration of Interests

None.
